# Supplementary material for: Prediction of Bacteremia Based on 12-Year Medical Data Using a Machine Learning Approach: Effect of Medical Data by Extraction Time
Source: Diagnostics (Basel). 2022 Jan 3;12(1):102. doi: 10.3390/diagnostics12010102 (PMC8774637; doi:10.3390/diagnostics12010102)
Supplement: Supplementary file 1 [file diagnostics-12-00102-s001.zip › diagnostics-1493813-supplementary.pdf]

Table S1. Top twenty bacterial strains identified in blood culture.

Rank	Pathogen	Number
1	*Escherichia coli*	9,261
2	*Staphylococcus aureus*	5,109
3	*Klebsiella pneumoniae*	4,709
4	*Enterococcus faecium*	4,577
5	*Staphylococcus epidermidis*	3,201
6	*Acinetobacter baumannii*	2,583
7	*Pseudomonas aeruginosa*	1,694
8	*Enterococcus faecalis*	1,366
9	*Enterobacter cloacae*	636
10	*Micrococcus spp.*	591
11	*Staphylococcus hominis*	475
12	*Bacteroides fragilis*	365
13	*Staphylococcus capitis*	348
14	*Enterobacter aerogenes*	344
15	*Staphylococcus haemolyticus*	327
16	*Klebsiella oxytoca*	311
17	*Serratia marcescens*	303
18	*Bacillus spp.*	281
19	*Citrobacter freundii*	247
20	*Proteus mirabilis*	236

Number: the number of times that these pathogens were cultured

Table S2. Baseline characteristic between bacteremia and non-bacteremia group using clinical data from 12-hours before blood culture tests.

	Total			Hospital A			Hospital B		
	**Bacteremia, Yes (n=77,357)**	**Bacteremia, No (n=260,691)**	***P*-value**	**Bacteremia, Yes (n=16,112)**	**Bacteremia, No (n=40,002)**	***P*-value**	**Bacteremia, Yes (n=61,245)**	**Bacteremia, No (n=220,689)**	***P*-value**
Age, years	60.1 ± 13.9	58.5 ± 14.7	<0.001	61.4 ± 13.4	58.2 ± 15.6	<0.001	59.8 ± 14.0	58.5 ± 14.6	<0.001
Sex, male	47,145 (60.1%)	154,642 (59.3%)	<0.001	9,938 (61.7%)	24,316(60.1%)	<0.001	37,207 (60.1%)	130,326 (59.0%)	<0.001
Vital Sign									
SBP, mmHg	123.0 ± 22.3	124.0 ± 21.5	0.052	121.1 ± 25.0	122.3 ± 23.9	0.055	123.6 ± 21.5	124.3 ± 21.0	<0.001
DBP, mmHg	70.0 ± 14.3	71.6 ± 13.7	<0.001	67.5 ± 14.3	70.2 ± 14.1	<0.001	70.6 ± 14.2	71.9 ± 13.6	0.021
Body temperature, °C	37.1 ± 0.7	37.1 ± 0.7	<0.001	36.9 ± 0.7	36.9 ± 0.7	0.089	37.1 ± 0.7	37.1 ± 0.7	<0.001
Heart rate, beats/min	95.2 ± 20.8	90.9 ± 19.5	<0.001	96.7 ± 21.3	92.5 ± 21.1	<0.001	94.8 ± 20.7	90.6 ± 19.2	<0.001
Respiratory rate, /min	20.2 ± 4.7	19.8 ± 4.1	<0.001	20.2 ± 5.3	19.3 ± 4.6	<0.001	20.2 ± 4.5	19.9 ± 4.0	<0.001
Hospital day, days	19.7 ± 33.0	10.6 ± 17.9	<0.001	21.1 ± 36.1	7.0 ± 13.4	<0.001	19.3 ± 32.1	11.2 ± 18.5	0.001
Laboratory data									
WBC, 10^3/μL	10.5 ± 9.2	10.4 ± 9.4	0.026	11.8 ± 7.9	11.4 ± 7.7	0.277	10.2 ± 9.5	10.2 ± 9.7	<0.001
Neutrophil, 10^3/μL	9.1 ± 7.3	8.5 ± 6.0	0.009	9.9 ± 7.3	9.1 ± 6.4	<0.001	9.0 ± 7.4	8.4 ± 5.9	<0.001
Monocyte, 10^3/μL	0.4 ± 0.4	0.5 ± 0.6	<0.001	0.5 ± 0.4	0.7 ± 1.1	<0.001	0.4 ± 0.3	0.5 ± 0.3	0.081
Hemoglobin, g/dL	9.6 ± 1.9	10.3 ± 2.1	<0.001	10.0 ± 1.9	10.9 ± 2.4	<0.001	9.5 ± 1.9	10.2 ± 2.0	<0.001
Platelet count, 10^3/μL	145.8 ± 128.4	208.6 ± 138.6	<0.001	176.5 ± 136.2	215.1 ± 127.4	<0.001	137.9 ± 125.1	207.3 ± 140.7	<0.001
ESR, mm/h	50.2 ± 37.1	52.7 ± 36.0	0.008	48.3 ± 35.4	46.6 ± 35.7	0.127	50.8 ± 37.6	53.9 ± 36.0	<0.001

CRP, mg/L	123.2 ± 95.9	84.3 ± 79.7	<0.001	128.5 ± 90.0	91.7 ± 86.6	<0.001	120.5 ± 98.7	81.8 ± 77.1	<0.001
Cholesterol, mg/dL	112.2 ± 48.6	127.5 ± 50.1	<0.001	111.6 ± 48.4	135.2 ± 52.8	<0.001	112.4 ± 48.7	124.8 ± 48.8	<0.001
Glucose, mg/dL	155.9 ± 100.3	146.7 ± 71.2	<0.001	155.4 ± 79.3	152.3 ± 87.9	0.028	156.1 ± 106.3	145.0 ± 65.4	<0.001
BUN, mg/dL	33.9 ± 25.3	24.0 ± 19.1	<0.001	34.0 ± 26.0	25.1 ± 20.2	<0.001	33.9 ± 25.1	23.8 ± 18.8	<0.001
Creatinine, mg/dL	1.5 ± 1.6	1.3 ± 1.6	<0.001	1.4 ± 1.4	1.4 ± 1.8	0.027	1.5 ± 1.6	1.3 ± 1.5	<0.001
Total protein, g/dL	5.4 ± 1.0	5.7 ± 1.0	<0.001	5.7 ± 1.0	6.0 ± 1.0	<0.001	5.4 ± 1.0	5.6 ± 0.9	<0.001
Albumin, g/dL	2.8 ± 0.6	3.0 ± 0.6	<0.001	2.9 ± 0.6	3.3 ± 0.7	<0.001	2.7 ± 0.6	3.0 ± 0.6	<0.001
Uric acid, mg/dL	4.3 ± 2.7	4.0 ± 2.4	0.007	4.1 ± 2.7	4.6 ± 2.6	<0.001	4.3 ± 2.7	3.8 ± 2.3	<0.001
ALP, IU/L	194.5 ± 247.6	129.2 ± 154.0	<0.001	210.0 ± 267.6	136.1 ± 161.8	<0.001	189.9 ± 241.2	127.2 ± 151.6	<0.001
AST, IU/L	204.0 ± 1024.9	122.9 ± 650.5	<0.001	178.1 ± 926.5	162.2 ± 877.1	<0.001	211.1 ± 1050.1	113.0 ± 579.6	<0.001
ALT, IU/L	102.1 ± 357.3	75.1 ± 283.1	<0.001	96.9 ± 334.5	98.2 ± 400.0	<0.001	103.6 ± 363.3	69.3 ± 244.8	<0.001
T. bilirubin, mg/dL	3.7 ± 6.0	1.8 ± 3.5	<0.001	3.7 ± 6.0	1.8 ± 3.5	<0.001	3.7 ± 5.9	1.7 ± 3.2	<0.001
Inorganic P, mg/dL	3.4 ± 1.6	3.3 ± 1.4	0.244	3.3 ± 1.4	3.5 ± 1.6	0.002	3.5 ± 1.6	3.3 ± 1.3	<0.001
Calcium, mg/dL	8.3 ± 0.9	8.3 ± 0.8	0.270	8.3 ± 1.0	8.3 ± 0.8	0.083	8.2 ± 0.9	8.3 ± 0.8	0.120
Sodium, mmol/L	137.6 ± 6.3	137.7 ± 5.6	0.001	137.9 ± 6.6	137.3 ± 6.0	0.067	137.5 ± 6.2	137.8 ± 5.5	<0.001
Potassium, mmol/L	3.9 ± 0.8	3.9 ± 0.7	<0.001	3.9 ± 0.7	4.0 ± 0.7	<0.001	3.9 ± 0.8	3.9 ± 0.7	<0.001
Chloride, mmol/L	101.2 ± 6.5	101.7 ± 6.0	<0.001	103.0 ± 6.6	102.8 ± 6.4	0.284	100.8 ± 6.4	101.4 ± 5.9	0.009
tCO2, mmol/L	22.7 ± 5.4	23.4 ± 4.7	<0.001	23.5 ± 5.4	23.5 ± 5.1	0.248	22.5 ± 5.4	23.3 ± 4.5	0.146
PT, sec	16.4 ± 9.6	15.0 ± 7.1	<0.001	17.1 ± 5.6	15.9 ± 7.8	<0.001	16.2 ± 10.3	14.8 ± 7.0	0.206
aPTT, sec	42.8 ± 21.4	38.8 ± 19.3	<0.001	46.7 ± 20.1	39.6 ± 18.1	<0.001	41.9 ± 21.6	38.6 ± 19.6	<0.001

Abbreviation, Hospital A, Sinchon Severance hospital; Hospital B, Gangnam Severance Hospital; SBP, systolic blood pressure; DBP, diastolic blood pressure; WBC, white blood cell; ESR, erythrocyte sedimentation rate; CRP, C-reactive protein; BUN, blood urea nitrogen; ALP, alkaline

phosphatase; AST, aspartate transaminase; ALT, alanine transaminase; T. bilirubin, total bilirubin; P, phosphate; PT, prothrombin time; aPTT, activated partial thromboplastin time.
